# Simultaneous MV‐kV imaging for intrafractional motion management during volumetric‐modulated arc therapy delivery*

**DOI:** 10.1120/jacmp.v17i2.5836

**Published:** 2016-03-08

**Authors:** Margie A. Hunt, Mark Sonnick, Hai Pham, Rajesh Regmi, Jian‐ping Xiong, Daniel Morf, Gig S. Mageras, Michael Zelefsky, Pengpeng Zhang

**Affiliations:** ^1^ Department of Medical Physics Memorial Sloan Kettering Cancer Center New York NY; ^2^ Weill Cornell Medical College New York NY; ^3^ Varian Medical Systems Palo Alto CA; ^4^ Department of Radiation Oncology Memorial Sloan Kettering Cancer Center New York NY USA

**Keywords:** image‐guided radiotherapy, motion monitoring, fiducial tracking

## Abstract

The purpose of this study was to evaluate the accuracy and clinical feasibility of a motion monitoring method employing simultaneously acquired MV and kV images during volumetric‐modulated arc therapy (VMAT). Short‐arc digital tomosynthesis (SA‐DTS) is used to improve the quality of the MV images that are then combined with orthogonally acquired kV images to assess 3D motion. An anthropomorphic phantom with implanted gold seeds was used to assess accuracy of the method under static, typical prostatic, and respiratory motion scenarios. Automatic registration of kV images and single MV frames or MV SA‐DTS reconstructed with arc lengths from 2° to 7° with the appropriate reference fiducial template images was performed using special purpose‐built software. Clinical feasibility was evaluated by retrospectively analyzing images acquired over four or five sessions for each of three patients undergoing hypofractionated prostate radiotherapy. The standard deviation of the registration error in phantom using MV SA‐DTS was similar to single MV images for the static and prostate motion scenarios (σ=0.25 mm). Under respiratory motion conditions, the standard deviation of the registration error increased to 0.7 mm and 1.7 mm for single MV and MV SA‐DTS, respectively. Registration failures were observed with the respiratory scenario only and were due to motion‐induced fiducial blurring. For the three patients studied, the mean and standard deviation of the difference between automatic registration using 4° MV SA‐DTS and manual registration using single MV images results was 0.07±0.52 mm. The MV SA‐DTS results in patients were, on average, superior to single‐frame MV by nearly 1 mm — significantly more than what was observed in phantom. The best MV SA‐DTS results were observed with arc lengths of 3° to 4°. Registration failures in patients using MV SA‐DTS were primarily due to blockage of the gold seeds by the MLC. The failure rate varied from 2% to 16%. Combined MV SA‐DTS and kV imaging is feasible for intratreatment motion monitoring during VMAT of anatomic sites where limited motion is expected, and improves registration accuracy compared to single MV/kV frames. To create a clinically robust technique, further improvements to ensure visualization of fiducials at the desired control points without degradation of the treatment plan are needed.

PACS number(s): 87.55.km, 87.55.N‐

## I. INTRODUCTION

The possibility of detecting and correcting for motion during delivery of external beam radiotherapy has become a realistic goal with the advent of sophisticated imaging, registration, and tracking technologies coupled to linear accelerators. A variety of methods for detecting and quantifying intratreatment motion have been developed, including those that rely on the implantation of radiofrequency beacons^1^, ^2^, ^3^, ^4^ and those that are based on the imaging of bony anatomy or implanted radiopaque fiducials.^5^, ^6^, ^7^, ^8^, ^9^, ^10^, ^11^, ^12^, ^13^, ^14^ For the most part, image‐based intratreatment motion detection has been studied using MV or on‐board (i.e., gantry‐mounted) kilovoltage (kV) single‐source systems^6^, ^9^, ^11^ or external (i.e., room‐mounted) dual‐kV source systems.^5^, ^13^, ^14^ Although kV systems provide high‐quality images, current capabilities related to image acquisition frequency and directionality of the images can limit three‐dimensional motion detection, either because the imaging system consists of a single kV source orthogonal to the MV beam direction (as is the case for accelerator on‐board systems) or because the patient field of view may periodically be occluded by the accelerator itself (for external dual‐source systems). Motion detection in two dimensions with gantry‐mounted kV systems is often less than ideal since the detected motion in the transverse direction of the image is typically along the direction of the MV beam and, therefore, of limited dosimetric usefulness. Combining simultaneously acquired intratreatment megavoltage images with kilovoltage images may offer some advantage over methods based on kV alone and has been studied by several groups.^7^, ^10^, ^15^, ^16^, ^17^, ^18^, ^19^ Liu et al.^(10)^ evaluated a combined MV‐kV approach for use during volumetric‐modulated arc therapy (VMAT) delivery and found an accuracy of 0.5 mm under conditions simulating normal adult breathing and 1.5 mm under more extreme motion conditions. Yan et al.^(19)^ reported a method whereby stereoscopic MV and kV imaging were used at the start of treatment to obtain 3D positions of implanted fiducials, but then the kV imaging was switched off except when the fiducials were not apparent in the MV images. In this method, the 3D position is estimated by combining the MV data with motion correlations established during the initial stereoscopic imaging period.

As pointed out in these and other studies, MV images acquired with an electronic portal imaging device (EPID) during treatment are “free” in terms of patient dose, provide information in the most relevant directions, and can be combined with kV images to obtain real‐time 3D patient position. Their principal limitations are relatively poor image quality and a field of view limited to the field aperture at any particular moment. The purpose of this study was to develop and investigate the feasibility of a new method for simultaneous acquisition of MV and kV images specifically for VMAT that would improve image quality over standard MV images and demonstrate accuracy for clinical evaluation of intratreatment patient motion. The method uses short‐arc digital tomosynthesis (SA‐DTS) to improve the quality of the MV images. Phantom studies were used to demonstrate MV SA‐DTS feasibility and to evaluate the accuracy of the combined MV‐kV approach. Once feasibility and accuracy were established, images were acquired for a small group of patients and retrospectively analyzed with the purpose of demonstrating clinical feasibility and accuracy.

## II. MATERIALS AND METHODS

### A. Phantom study

The simultaneous MV‐kV imaging technique was developed on a Varian TrueBeam linear accelerator equipped with an on‐board kilovoltage imaging system with a high frame rate amorphous silicon (aSi) flat‐panel imager (40×30 cm, 0.388 mm effective pixel size), a PortalVision aS1000 megavoltage EPID (40×30 cm, 0.392 mm effective pixel size), and a 120 leaf Millenium multileaf collimator (MLC) (Varian Medical Systems, Palo Alto, CA). The technique was developed specifically for application during VMAT since the somewhat larger MLC apertures improve the likelihood of fiducial visualization on the MV images compared to traditional IMRT delivered with the sliding window technique.

Phantom studies were performed to evaluate the technique accuracy, defined as the standard deviation of the difference between the planned and measured phantom positions under different motion scenarios. Three gold seed cylindrical fiducials (Mick Radio‐Nuclear Instruments, Mt. Vernon, NY) (3 mm×1.2 mm) were inserted into an anthropomorphic RANDO phantom (The Phantom Laboratory, Salem, NY) near vertebral bodies in the lower thoracic spine region. Although ultimately, we applied our technique to intratreatment imaging during prostate treatments, the method could be considered for any anatomic site for which fiducials can be implanted. Hence, we chose what we felt was a generally representative anatomic location for the phantom study.

**Figure 1:**
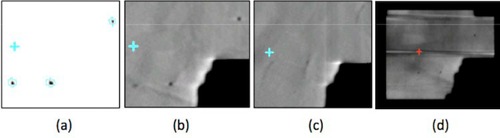
Fiducial template and MV images. (a) Fiducial template created using the method of Regmi et al.^(20)^ Blue contours indicate the regions of interest for computing the autoregistration cost function (see text). (b) Five‐frame MV SA‐DTS image under no‐motion conditions. (c) Five‐frame MV SA‐DTS under respiratory motion conditions. (d) MV image after processing to remove bands cause by asynchrony between the detector readout and beam pulses.

A planning CT of the phantom with slice spacing of 2 mm was obtained and used to generate 2D reference images at one degree gantry angle increments using a modification to the template technique described by Mostafavi et al.^(12)^ and implemented at our institution by Regmi et al.^(20)^ Briefly, generation of the reference template images involved initial segmentation of the fiducials on the planning CT, followed by the addition of a 1 mm margin to create a region of interest (ROI). In the modified version, the center‐of‐mass and orientation of each fiducial was then determined and used to position a template of the physical shape of the each fiducial within each ROI. Lastly, a final projection of the ROI into the image plane was used to create the reference template images. The projected ROIs define the regions over which the autoregistration cost function is computed (described below). An example of a reference template image of the fiducials and ROIs is shown in Fig. 1(a), demonstrating the improved image quality compared to standard DRRs, thereby reducing uncertainty in the subsequent registration of the treatment and reference images. To account for changes in the relative marker positions between simulation and treatment (e.g., owing to tissue deformations or fiducial migration), the fiducial position in the template images can be adjusted based on an orthogonal kV image pair during patient setup.^(20)^

For the phantom study, a single 360° 6 MV prostate VMAT arc centered on the fiducials, with 177 control points, constant gantry speed, and limited MLC motion (MLC motion was similar to conformal arc) was created. MLC motion was kept to a minimum so as to minimize the likelihood of MLC interference with fiducial visualization. The phantom studies were carried out within the TrueBeam Developer mode (Version 1.5) by converting the plan control points into custom XML (extensible markup language) files and inserting additional commands to acquire kV and MV images during the VMAT delivery. kV imaging was specified in the XML file using the dynamic gain fluoro‐acquisition mode which acquires images continuously throughout the arc delivery at ~11 Hz. Since it is not currently possible to specify simultaneous continuous intratreatment acquisition of MV and kV images in either developer or clinical mode on TrueBeam, proprietary software, iTools Capture, provided by Varian through a research agreement, was used to passively grab MV images from the EPID in the high‐resolution mode at ~9.5 Hz during treatment delivery and kV images at ~11 Hz. These images were in the Varian proprietary .XIM file format, but lacked image header information and could only be read using software also provided by Varian. In particular, at the time the images were acquired, the lack of MV image header information made it difficult to accurately establish the image acquisition angle. To overcome this, we used the constant gantry rotation speed during the VMAT delivery and the known MV frame rate to assign initial gantry angles to each image. Verification and subsequent modification of the MV gantry angles was done by manually comparing the MLC aperture in a subset of the MV images to the planned aperture. We estimate the inaccuracy of the MV gantry angle assignment as a result of this process to be approximately 2°, leading to an inaccuracy in the determination of the 2D and subsequent 3D position (as described in Appendix A) of less than 0.05 mm. Each VMAT delivery took slightly more than 1 min and yielded approximately 670 kV and 580 MV images. Each kV image was paired with the MV image closest to kV orthogonality to create an MV‐kV pair, leading to a temporal and angular resolution of approximately 0.1 s and 0.6°.

The ability to visualize and register fiducials in individual MV frames was limited under any of following conditions: low‐dose segments, asynchronous readout of the detector with respect to the beam pulses resulting in horizontal bands in the image, significant overlying anatomy, and obstruction of the fiducials by the MLC. Removal of the horizontal bands from each MV image was achieved with the following process: pixels in the irradiated region (i.e., exposed by the MLC apertures) were identified by thresholding of the intensity histogram of the image. Next, the average intensity $I_{\text{ave},r}$ of the irradiated pixels in each row, r, of the image was computed and the maximum row intensity $I_{\text{ave},\text{max}}$ was determined. For each row of the image, the intensity of each irradiated pixel was then adjusted by multiplying it by a factor $I_{\text{ave},\text{max}}/I_{\text{ave},r}$. To reduce the impact of overlying anatomy, we developed a method using short‐arc digital tomosynthesis (SA‐DTS) based on a multiple projection algorithm, as described by Kolitsi et al.^(21)^ (Fig. 1). This method offers the advantage of selective removal of out‐of‐plane objects which can diminish visualization of bony anatomy, leading to image enhancement for better detection and localization of fiducials.^(22)^

Registration of the MV SA‐DTS and kV images to the reference fiducial template images was performed using purpose‐built registration software.^(20)^ For each individual MV and kV image, the reference image closest in angle was selected and 2D template‐based autoregistration was performed using simplex‐based minimization of a normalized cross‐correlation cost function. In order to avoid trapping in a local minimum, a grid of starting positions was defined within the search region (10×10 mm initially; 5×5 mm on subsequent images) with 2 mm grid spacing. The cost was calculated at each grid point and the five points with the lowest costs were selected. Simplex minimization was started at each of these points, and the minimization with the lowest cost was selected (subject to satisfying the figure‐of‐merit criterion, described below). The resultant match position served as the initial starting point for registration of the next image; in addition, the registration was repeated starting from the planned position, and the match position with the lower cost was selected. The latter registration allowed the tracking procedure to recover from occasional misregistrations that resulted in the fiducials lying outside the search region of the subsequent image. To further improve the robustness of the registration, we incorporated a figure‐of‐merit (FOM) described by Mostafavi et al.^(22)^ that utilizes a peak‐to‐sidelobe ratio as a measure of MV SA‐DTS fiducial detectability. The peak‐to‐sidelobe ratio is defined as the cost at the registration match point (peak) divided by the standard deviation of the cost at points in the vicinity (”sidelobe”) away from the peak. For each registration, the FOM at the match point is compared to a threshold determined empirically from evaluating phantom images with differing image noise and fiducial visibility. In the event the FOM falls below the threshold (FOM failure), the registration reverts to a 2D kV‐only assessment and stops to alert the user who can then modify the registration result or allow the process to continue to the next image pair. Throughout this study, each FOM failure was noted, but no correction was made to the registration result prior to resuming. Registration results were presented as the difference in the center‐of‐mass of the fiducials on the MV or kV image and the reference template image. The 2D center‐of‐mass positions for each MV‐kV pair were then used to determine the 3D position using a method similar to that described by Liu et al.^(10)^ and summarized in Appendix A.

The accuracy of this combined MV SA‐DTS and kV technique, defined by standard deviation of the difference between the planned and measured phantom positions, was evaluated under scenarios of static, typical prostatic motion, and respiratory motion. For each motion scenario, the anthropomorphic phantom was positioned on the treatment couch as close to the known position as possible using pretreatment orthogonal kV imaging matched to standard DRRs. For the static evaluation, the VMAT arc was then delivered from Developer mode acquiring MV and kV images as described above. To simulate prostate motion, translations from a typical Calypso (Varian Medical Systems) trace with maximum excursions in the vertical, lateral, and longitudinal directions of 0.19, 0.18, and 0.49 cm, were converted to couch positions at each of the 177 VMAT control points and embedded into the XML file. To simulate respiratory motion, a periodic but asymmetric function allowing for additional time spent in the end‐exhale position^(23)^ was applied with a 4 s period and peak‐to‐peak amplitudes of 0.5, 0.2, and 1.0 cm in the vertical, lateral, and longitudinal directions. For each “motion incorporated” VMAT delivery, phantom motion was achieved through continuous couch motion. This method allowed us to study accuracy using an anthropomorphic phantom and also to reliably establish the couch (and therefore phantom) position at each control point.

To determine the value of using MV SA‐DTS compared to MV images alone, registration error using kV combined with either single MV images or MV SA‐DTS reconstructed over arc lengths varying from 2° to 7° (3 to 11 frames) was evaluated. For each motion scenario and MV/kV combination (i.e., MV SA‐DTS or single MV image), the mean and standard deviation of the difference between the measured and planned phantom position for each image pair‐was determined. The goal of this part of the study was to evaluate differences in accuracy and registration failure rate for different MV SA‐DTS angular lengths and to determine limitations of the technique in the presence of motion.

### B. Patient studies

**Figure 2:**
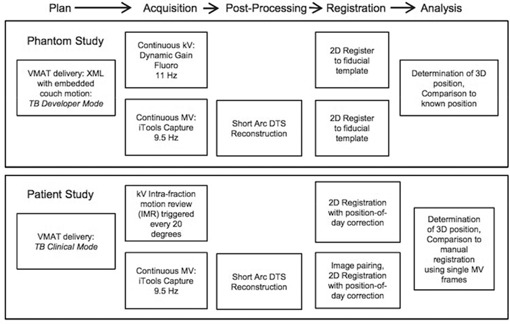
Simultaneous MV/kV imaging process for phantom and patient studies.

Once phantom studies were complete and appropriate IRB approval was obtained, clinical feasibility of simultaneous MV‐kV imaging was studied by retrospectively analyzing images for three patients who underwent hypofractionated (5‐fraction) VMAT prostate radiotherapy to 40 Gy. Simultaneous MV‐kV imaging was done for each fraction but used solely for retrospective QA. Each patient had three intraprostatic gold seed fiducials implanted and was treated with two full VMAT arcs in TrueBeam clinical mode with collimator angles of either 0° and 90° or 315° and 45°. As part of each patient's prescribed treatment, daily pretreatment 2D kV orthogonal images were obtained with matching based on the gold seed locations. Intratreatment kV imaging triggered every 20° was then obtained using the intrafraction motion review (IMR) functionality on the TrueBeam. iTools Capture was used to passively grab the triggered kV images, as well as the MV images acquired continuously throughout delivery at ~9.5 Hz. Image registration and 3D patient position determination were similar to that described for the phantom study. The only significant difference was that an attempt was made to minimize systematic registration error due to day‐to‐day fiducial deformation by correcting the fiducial positions in the reference images to a “position of the day” based on their observed positions in the daily pretreatment kV pair.^(20)^ A schematic of the treatment and image analysis process for the phantom and patient studies is given in Fig. 2. Analysis of the patient images was done by comparing the mean and standard deviation of the difference between motion detected using manual registration with single MV frames to that obtained with automatic registration using single MV frames or MV SA‐DTS with arc lengths of 2° to 7°. In addition, the frequency of FOM failures was recorded.

## III. RESULTS

### A. Phantom studies

**Figure 3:**
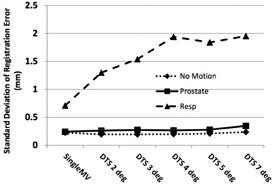
SD of the registration error (measured ‐ planned position) for different motion scenarios using kV imaging combined with either single MV frames or MV SA‐DTS reconstructed from arc lengths of 2° to 7°. Results are averaged over the three directions.

For each phantom motion scenario, approximately 660 MV‐kV pairs were available for analysis. The standard deviations (SDs) of the registration error averaged over all three directions for the different motion scenarios and MV image types analyzed are shown in Fig. 3. The results using kV combined with single MV frames versus MV SA‐DTS reconstructed over different arc lengths were very similar for the static and prostate scenarios. The standard deviation for the static scenario was 0.22 mm for single MV frame registration and varied from 0.19 mm for 2° MV SA‐DTS to 0.24 mm for 7° MV SA‐DTS. For prostate motion, single MV frame registration was 0.24 mm and MV SA‐DTS ranged from 0.26 mm for 2° reconstruction to 0.34 mm for 7° reconstruction. In the case of respiratory motion, the results were significantly worse for all image types, but single MV fared better than MV SA‐DTS. Single‐frame MV yielded a standard deviation of 0.7 mm, while MV SA‐DTS ranged from 1.30 for 2° reconstruction to 1.95 mm for 7° reconstruction. Furthermore, while there were no FOM failures for the static and prostate motion scenarios when using either single MV or MV SA‐DTS, there were four failures with respiratory motion and single‐frame MV and as many as 12 failures with MV SA‐DTS reconstructions between 3° and 7° as a result of motion‐induced fiducial blurring.

**Figure 4:**
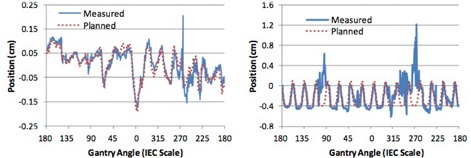
Planned and measured phantom positions using the combined MV SA‐DTS (4° reconstruction) and kV technique in the anterior‐posterior direction for the (a) prostate and (b) respiratory motion scenarios.

The mean and standard deviation of the registration error in each direction using single frame MV and 4° MV SA‐DTS for all three motion scenarios is given in Table 1. Results were similar using either single MV or MV SA‐DTS for the static and prostate motion scenarios with standard deviations ranging from approximately 0.1 to 0.3 mm. The standard deviation of the registration error was approximately 0.1 mm less in the superior‐inferior direction using either single MV or MV SA‐DTS for the static scenario, but increased to approximately 0.2 mm, similar to the other directions, with the prostate motion scenario. Representative traces for the prostate and respiratory motion scenarios using 4° MV SA‐DTS are shown in Fig. 4.

**Table 1:** Phantom 3D registration results using kV imaging combined with single MV or MV SA‐DTS

Measured−Planned Position (mm) (Mean±SD)						
	*Left‐Right*		*Ant.‐Post*.		*Sup.—Inf*.	
*Motion Scenario*	*Single MV*	*MV SA‐DTS (4°)*	*Single MV*	*MV SA‐DTS (4°)*	*Single MV*	*MV SA‐DTS (4°)*
Static	−0.35±0.25	−0.32±0.21	0.40±0.27	0.34±0.25	0.29±0.13	0.36±0.12
Prostate Motion	−0.03±0.27	−0.05±0.32	−0.60±0.24	−0.63±0.26	0.36±0.21	0.34±0.21
Respiratory Motion	0.03±0.48	−0.03±1.18	−0.51±0.73	0.32±2.27	0.28±0.87	−1.02±2.17

### B. Patient studies

**Figure 5:**
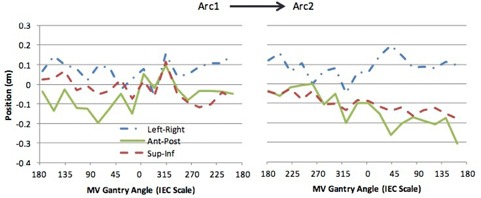
Representative traces obtained for a patient undergoing hypofractionated VMAT prostate radiotherapy. The traces were generated from combined kV and 4° MV SA‐DTS imaging.

Images from four treatment sessions were available for two patients and five sessions for one patient with 19 MV/kV pairs from each of the two arcs for each session, yielding a total of 38 image pairs per session and 494 image pairs in total. An example of 3D motion obtained during a treatment session and analyzed using kV and MV SA‐DTS (4° reconstruction) is shown in Fig. 5 for a patient who experienced significant drift of the prostate in the posterior‐inferior direction over the course of the treatment session.

**Figure 6:**
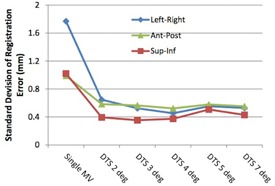
SD of the registration error averaged over all treatment sessions for a patient undergoing hypofractionated prostate radiotherapy. Results are shown for kV imaging combined with either single MV frames or MV SA‐DTS reconstructed from arc lengths of 2° to 7°.

To validate the accuracy of the MV SA‐DTS and kV method for the patient study, the intratreatment motion determined using autoregistration with kV and single MV frames or MV SA‐DTS reconstructed with arc lengths from 2° to 7° was compared to manual registration using kV and single MV frames, representing ground truth. The standard deviation of the difference between manual and autoregistration for one of the patients is shown Fig. 6, demonstrating the improvement for patients obtained with MV SA‐DTS compared to single‐frame MV. The standard deviation of the difference between manual and autoregistration using single MV frames, averaged over the three patients, was 1.55 mm, while for MV SA‐DTS it was 0.60 mm with a minimum of 0.52 mm for 4° MV SA‐DTS. The number of registration failures also differed when single‐frame MV or MV SA‐DTS was used. Autoregistration with single‐frame MV resulted in an average of 9 FOM failures for all sessions combined, leading to an overall registration success rate of 94%. With MV SA‐DTS, the average FOM registration success rate was 91%−93% for reconstruction arc lengths less than 5°. For larger arc lengths, the registration success rate decreased significantly to an average of 87% and 73% for 5° and 7° MV SA‐DTS, respectively. This was due to more instances where the fiducials were blocked by the MLC as a result of the longer arc length, and was most evident for VMAT arcs with a collimator angle of 90° which had a high level of MLC modulation. Overall, the standard deviations of the difference between manual single frame MV and autoregistration using 4° MV SA‐DTS were similar for all three patients (Table 2) and ranged from 0.37 to 0.66 mm. The VMAT arcs for Patient 2 appeared to have more MLC modulation, leading to generally higher standard deviations and a significantly lower registration success rate.

**Figure A1:**
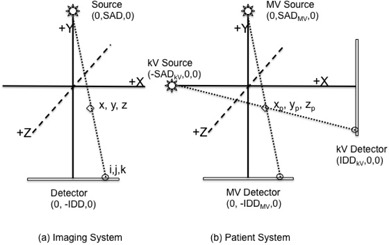
Coordinate systems used in the determination of 3D position. All three systems (MV, kV, and patient) have the origin defined at the machine isocenter. The MV and kV imaging systems are shown at their home positions (Gantry 0) in (b).

The intratreatment motion from this small group of patients was evaluated by finding the mean motion during each session (using kV with 4° MV SA‐DTS) and then averaging over all sessions. The mean displacement ranged from 0.12 to 1.4 mm across all directions and the standard deviation ranged from 0.47 to 1.9 mm. The largest motion was observed in the posterior and inferior directions. Patients 1 and 2 experienced at least one occurrence of motion exceeding 2 mm in any direction in three of the four treatment sessions evaluated, while Patient 3 experienced such motion in four of five treatment sessions. The maximum displacement during a single session was 2.56 mm for Patient 1 (lateral direction), 3.91 mm for Patient 2 (AP direction), and 7.04 mm for Patient 3 (AP direction). These results appear similar to those obtained in other studies using other methods.^2^, ^24^

**Table 2:** Mean and SD of the difference between manual registration using kV and single MV frames and automatic registration using kV and 4° MV SA‐DTS in patients

*Patient*	*Left‐Right (mm)*	*Ant.‐Post. (mm)*	*Sup.—Inf. (mm)*	*% Reg. Success*
1	0.01±0.45	0.05±0.52	0.00±0.37	97.8
2	0.11±0.63	0.11±0.59	0.23±0.59	83.8
3	−0.04±0.42	0.11±0.66	0.04±0.38	91.8

% Reg.Success=the frequency of image pair registrations without FOM failures.

## IV. DISCUSSION

The introduction of kilovoltage imaging systems, mounted orthogonal to the treatment head, has significantly improved both inter‐ and intratreatment motion monitoring. These systems have become commonplace to support improved pretreatment radiographic and cone‐beam CT imaging, determination and correction of 3D position, and reduced margins. Although the use of kV systems to monitor intratreatment motion is expanding, the determination of 3D position using an on‐board kV system alone is not straightforward.^25^, ^26^ Currently, intratreatment kV monitoring is usually limited to 2D position determination orthogonal to the beam direction, but such approaches need to be used with caution since they are likely to miss detection of at least some of the motion in the dosimetrically relevant BEV direction.

In this study, we present a method combining MV and kV to determine 3D position during intratreatment motion monitoring. The advantages of combined MV/kV imaging include its relative simplicity, direct visualization of motion from the beam's‐eye‐view perspective, improved accuracy compared to kV alone, and the potential to monitor 2D motion using MV alone so long as fiducials are visualized. To enhance the visualization of fiducials on the MV images, we investigated the use of MV digital tomosynthesis. The use of MV DTS as a substitute for either portal imaging or MV cone‐beam CT has been studied by Pang et al.^(27)^ who developed a pretreatment MV DTS method with arc length of 10°. Application of their method to intertreatment imaging of prostate patients demonstrated qualitatively that MV DTS improved fiducial visualization compared to portal imaging but was inferior to MV CBCT in terms of visualization of soft tissue. In our study, we have substantially decreased the arc length for the MV DTS in an attempt to maximize temporal resolution while still improving fiducial visualization over standard MV imaging.

In our phantom studies, we evaluated MV SA‐DTS arc lengths of 2° to 7° and found that, for the static scenario, the standard deviation of the registration error regardless of arc length was within 0.01 mm of that obtained using single MV images. For the prostate scenario, the standard deviations were within 0.02 mm of that obtained with single MV images for arc lengths less than 5°. With the largest MV SA‐DTS arc length (7°), an increase in the standard deviation of the registration error was observed (0.1 mm), most likely due to motion‐induced fiducial blurring. This degradation in results with MV SA‐DTS became much more apparent with respiratory motion. The standard deviation of the registration error with respiratory motion was 0.7 mm using single MV frames, but increased to 1.3 mm with 2° MV SA‐DTS and to 1.95 mm with 7° MV SA‐DTS. In addition to this loss in accuracy, four FOM failures were observed with the single MV frame registration and 12 with the larger MV SA‐DTS arc lengths. Taken together, we believe our phantom results indicate that registration using MV SA‐DTS with arc lengths less than 5° is superior to our simple approach using single MV frames and is suitable in situations with limited motion, such as during the delivery of prostate radiotherapy. However, MV SA‐DTS does not appear suitable in its current state for intratreatment monitoring of thoracic and abdominal sites.

Our analysis indicates a larger advantage to using MV SA‐DTS in patients than in phantom likely due to more complex and pronounced patient anatomy. MV SA‐DTS with arc lengths of less than 5° resulted in FOM failure rates in patients similar to single MV frames (8% versus 6%), but standard deviations of registration errors that were approximately 1 mm less. The results obtained with an MV SA‐DTS arc length of 4° appeared to offer the best compromise in terms of registration accuracy and FOM failure rate; however, optimal arc length is likely related to the amount of MLC modulation and may therefore vary with VMAT field design.

Several groups have described other approaches for simultaneous MV/kV imaging along with information on accuracy and robustness. Wiersma et al.^(18)^ reported on an early method of simultaneous MV/kV imaging which used an intensity‐based detection and registration method with a lag time of 150−200 ms. The RMS registration error for a pelvic phantom with periodic motion of 2 cm was less than 0.86 mm in each individual direction and 0.63 mm overall. Mao et al.^(16)^ described a method based on marker orientation and pattern matching to detect and register spherical and cylindrical fiducials within 100 ms. They reported standard deviations ≤0.5 mm using a static head phantom and an accuracy of 0.8 mm using a pelvic phantom under motion conditions.^(15)^ The fiducial detection success rate was 100% in an open field. Park et al.^(17)^ used a Laplacian filter to aid in fiducial detection coupled with projection of the fiducials from CT. Their 3D RMS registration error under static conditions in a Solid Water phantom was 0.43 mm with a detection success rate of 100% with static, open fields and a processing time of 9 to 15 frames/s. Their analysis of five patients undergoing SBRT liver treatment yielded 2D RMS errors (compared to manual registration) of approximately 0.75 mm.

The primary advantage of MV SA‐DTS over previously reported methods is the ability to selectively remove out‐of‐plane objects from the image, thereby enhancing fiducial visibility particularly for anatomical sites with complex overlying anatomy. Overall, our MV SA‐DTS method provides registration results in phantom and patients similar to those reported by others and discussed above. The standard deviation of our registration errors using 4° MV SA‐DTS for static and prostate motion scenarios in phantom did not exceed 0.32 mm and, in patients, it was no more than 0.66 mm. This 0.3 mm deterioration in MV SA‐DTS results when moving from phantom to patients is similar to that seen in the 2D results of Park et al.^(17)^ Both studies highlight the importance of evaluating imaging methodologies in patients in addition to phantom.

Our technique has several limitations that must be addressed prior to routine clinical implementation. Firstly, the total processing time for MV fiducial detection using MV SA‐DTS with a 4° arc length is currently 1.1 s (Intel Xeon E5620 2.4 GHz workstation), of which 0.6 s is the MV SA‐DTS reconstruction time. The processing time is slightly longer than the MV image acquisition time of 0.73 s over a 4.4° arc at a gantry rotation speed of 1 rpm (7 frames at 9.5 Hz acquisition rate). Although this processing time is significantly longer than that reported by others, it could be significantly shortened by parallelizing the algorithm and should not limit the ability to use the technique for real‐time motion monitoring.

Our patient study also highlighted a significant robustness limitation due to blockage or near‐blockage of the fiducials by the MLC aperture. The registration failure rate for the MV SA‐DTS varied from 2% to 16% for the three patients. This failure rate was significantly higher than that for the static and prostate motion phantom studies that were performed with simplistic VMAT MLC motion designed specifically to minimize MLC aperture conflicts. Determination of 3D position is possible as long as at least one fiducial is visualized in both images. However, in highly modulated VMAT deliveries, all fiducials may be blocked a significant amount of the time. To address this issue, we are developing the following approach as an initial step.^(28)^ At each control point at which an MV SA‐DTS/kV pair will be acquired, the VMAT MLC aperture is modified if needed to ensure visualization of at least one fiducial with margin sufficient to cover typical motion. A new dose calculation is performed with the modified apertures and compared to the original plan. Treatment proceeds as long as the new plan is considered acceptable. Our preliminary investigations indicate that, if imaging is performed every 20°, MLC aperture modification will be required for approximately 60% of the apertures and the resulting plan degradation will be acceptable in approximately 50% of patients. For the remaining patients, reoptimization, keeping the modified apertures fixed, results in a recovery of the original DVH characteristics.

Another area for future improvement in our technique is related to the creation of optimized MV SA‐DTS/kV imaging scenarios for individual patients. We have previously developed^(29)^ a method to design imaging scenarios based on the sensitivity of a patient's plan to motion. With such a scenario, the treatment delivery can be divided into bins of equal motion sensitivity with imaging performed at the start of each bin. An imaging method that incorporates MV SA‐DTS has much to offer in such an approach. Prior to treatment, all VMAT MLC apertures could be assessed for fiducial visualization with the intention of performing 2D MV SA‐DTS imaging at all suitable points. Since the 2D MV SA‐DTS data provides information in the BEV direction, the addition of the kV data, while valuable, might only be needed at a lesser frequency. Combined MV SA‐DTS and kV imaging with 3D position determination could be performed at the start of each sensitivity bin, thereby supplementing the previously acquired 2D data.

## V. CONCLUSIONS

Combined short arc MV DTS and kV imaging is feasible for intratreatment motion monitoring during VMAT of anatomic sites where limited motion is expected. The technique is superior to using standard single‐frame MV images and is capable of accurately detecting motion to within 1 mm. Furthermore, it may offer the potential benefit of 2D monitoring using MV SA‐DTS during all treatment segments when at least one fiducial is clearly visible. To create a clinically robust methodology, further methods to improve registration robustness and ensure optimal fiducial visualization at the desired control points without degradation to the treatment plan are needed.

## ACKNOWLEDGMENTS

This research was funded in part through the NIH/NCI Cancer Center Support Grant/Core Grant P30 CA008748.

## COPYRIGHT

This work is licensed under a Creative Commons Attribution 4.0 International License.

## APPENDICES

### Appendix 1. Derivation of 3D Coordinates from MV Short‐arc DTS and kV Projection Images

Three fixed coordinate systems for the patient, MV, and kV imaging systems were defined, each with its origin at the isocenter, as depicted in Fig. A1. For each imaging system, phantom points (X), the MV and kV sources (S), detectors (D), and their projections onto the image detectors (I) are as defined below:

(A.1) $$\begin{array}{l} \vec{X_{kV}}={\left[x_{kV},y_{kV},z_{kV}\right]}^{T}\;\text{and}\;\vec{X_{MV}}={\left[x_{MV},y_{MV},z_{MV}\right]}^{T}\\ \vec{S}={\left[0,SAD,0\right]}^{T}\;\text{and}\;\vec{D}={\left[0,-DD,0\right]}^{T}\\ \vec{I_{kV}}={\left[i_{kV},-DD,k_{kV}\right]}^{T}\;and\;\vec{I_{MV}}={\left[i_{MV},-DD,k_{MV}\right]}^{T} \end{array}$$

where *SAD* is the source‐axis distance (100 cm) and *IDD* is the isocenter‐detector distance (50 cm). Points in the kV or MV system, ($\vec{X_{kV}}$ or $\vec{X_{MV}}$), are related to the same point in the patient system, $\vec{X_{p}}$, and to each other by:

(A.2) $$\vec{X_{kV}}=r_{kV}\vec{X_{p}}$$

(A.3) $$\vec{X_{MV}}=r_{MV}\vec{X_{p}}$$

(A.4) $$\vec{X_{kV}}=r_{kV}r_{MV}{}^{T}\vec{X_{p}}$$

where $r_{\text{kV}}$ and $r_{\text{MV}}$ are rotation matrices given by:

(A.5) $$r\left[\begin{array}{lll} \text{cos}\vartheta & \text{sin}\vartheta & 0\\ -\text{sin}\vartheta & \text{cos}\vartheta & 0\\ 0 & 0 & 1 \end{array}\right]$$

and θ is the rotation angle of either the MV or kV system with respect to the patient system. The relationship between arbitrary points in the kV or MV system, $\vec{X_{kV}}$ or $\vec{X_{MV}}$, and their projections onto the corresponding image planes, $\vec{X_{kV}}$ or $\vec{X_{MV}}$, is given by

(A.6) $$\begin{array}{l} \vec{X_{kV}}=\frac{SAD_{kV}-y_{kV}}{SID_{kV}}(\vec{I_{kV}}-\vec{S_{kV}})+\vec{S_{kV}}\\ \;\;\;\;\;\;=\frac{SAD_{kV}-y_{kV}}{SID_{kV}}\left(\left[\overset{i_{kV}}{\underset{k_{kV}}{-DD_{kV}}}\right]-\left[\overset{0}{\underset{0}{SAD_{kV}}}\right]\right)+\left[\overset{0}{\underset{0}{SAD_{kV}}}\right] \end{array}$$

(A.7) $$\begin{array}{l} \vec{X_{mV}}=\frac{SAD_{MV}-y_{MV}}{SID_{MV}}(\vec{I_{MV}}-\vec{S_{MV}})+\vec{S_{MV}}\\ \;\;\;\;\;\;=\frac{SAD_{MV}-y_{MV}}{SID_{MV}}\left(\left[\overset{i_{MV}}{\underset{k_{MV}}{-DD_{MV}}}\right]-\left[\overset{0}{\underset{0}{SAD_{MV}}}\right]\right)+\left[\overset{0}{\underset{0}{SAD_{MV}}}\right] \end{array}$$

where *SID* is the source‐detector distance (150 cm). To obtain the coordinates of a fiducial, $\vec{X_{p}}$ in the patient system, we first determine the Y coordinates of the fiducial in the image systems ($y_{\text{kV}}$, $y_{\text{MV}}$) by combining (A.4), (A.5), (A.6), (A.7) above and applying least squares optimization (MATLAB, MathWorks, Natick, MA) to the resulting overdetermined problem:

(A.8) $$\begin{array}{l} \left[\begin{array}{lll} \frac{-i_{kV}}{SID} & \frac{\text{cos}(\vartheta -\vartheta_{MV})i_{MV}}{SID} & -\text{sin}(\vartheta_{kV}-\vartheta_{MV})\\ 1 & \frac{\text{sin}(\vartheta_{MV}-\vartheta_{kV})i_{MV}}{SID} & -\text{cos}(\vartheta_{kV}-\vartheta_{MV})\\ \frac{-k_{kV}}{SID} & \;\;\;\;\;\;\frac{k_{MV}}{SID} & \end{array}\right]\left[\begin{array}{l} y_{kV}\\ y_{MV} \end{array}\right]\\ \;\;\;\;\;\;\left[\begin{array}{c} \frac{SAD}{SID}(\text{cos}(\vartheta_{kV}-\vartheta_{MV})i_{MV}-i_{kV})\\ \frac{SAD}{SID}\text{sin}(\vartheta_{MV}-\vartheta_{kV})i_{MV}\\ \frac{SAD}{SID}(k_{MV}-k_{kV}) \end{array}\right] \end{array}$$

Once solved, the coordinates of the fiducial in the patient system, $\vec{X_{p}}$, are easily obtained from Eq. (A.2) or Eq. (A.3).

## Supporting information

Supplementary Material Files

Supplementary Material Files

Supplementary Material Files
