# Supplementary figures and images for: Simultaneous MV‐kV imaging for intrafractional motion management during volumetric‐modulated arc therapy delivery*
Source: J Appl Clin Med Phys. 2016 Mar 8;17(2):473–86. doi: 10.1120/jacmp.v17i2.5836 (PMC4831078; doi:10.1120/jacmp.v17i2.5836)

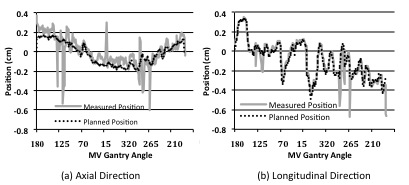

Supplement: Supplementary file 1 — Supplementary Material Files [file ACM2-17-473-s001.jpg]

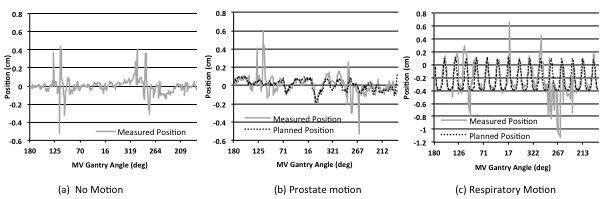

Supplement: Supplementary file 2 — Supplementary Material Files [file ACM2-17-473-s002.jpg]

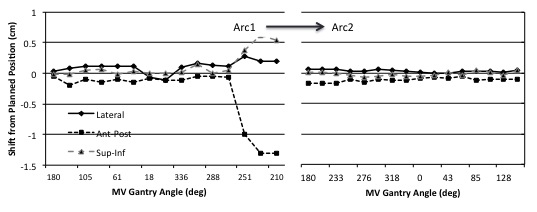

Supplement: Supplementary file 3 — Supplementary Material Files [file ACM2-17-473-s003.jpg]
